# Supplementary material for: The PATIENT Approach: A New Bundle for the Management of Chronic Pain
Source: J Pers Med. 2023 Oct 29;13(11):1551. doi: 10.3390/jpm13111551 (PMC10672627; doi:10.3390/jpm13111551)
Supplement: Supplementary file 1 [file jpm-13-01551-s001.zip › jpm-2647911-supplementary.pdf]

Table S1. Search Stretegy with results until to September 2022

Bundle	Query	Results in the screened databases	Included
**P- Patient's perception**	(chronic pain) AND (perception[Title]) AND ((age) OR (sex) OR (gender) OR (weight) OR (obesity) OR (sleep) OR (mood) OR (depression) OR (sexual abuse) OR (psychological status) OR (mental status) OR (anxiety))	271	15
**A- Assesment**	(((((pain[Title]) OR (assessment[Title])) OR (evaluation[Title])) AND (chronic pain[Title])) AND (((((nociceptive) OR (nociplastic)) OR (physiscal examination)) OR (maneuvers)) OR (scale))) AND (((shoulder) OR (back pain)) OR (knee))	429	9
**T- Tailored approach**	(pain[Title]) AND (chronic pain[Title/Abstract]) AND ((ladder[Title/Abstract]) OR (pharmacological therapy[Title/Abstract]) OR (minimally invasive[Title/Abstract]) OR (behavioural therapy[Title/Abstract]) OR (surgical therapy[Title/Abstract]) OR (physical therapy[Title/Abstract]) OR (drugs[Title/Abstract]) (opioids[Title/Abstract]) or (antiepiletic[Title/Abstract]) OR (gabapentinoids[Title/Abstract]) OR (nsaid[Title/Abstract]) OR (non-steroidal anti-inflammatory drugs[Title/Abstract]) OR (organ imparment[Title/Abstract]) OR (gene therapy[Title/Abstract]) OR (antidepressant[Title/Abstract]))	1202	13
**I- Iterative evaluation**	((((pain[Title]) AND (chronic pain[Title/Abstract]))) AND ((efficacy) OR (therapy))) AND (telemedicine[Text Word])	149	4
**E- Education**	(((pain[Title/Abstract]) AND ((education[Title/Abstract]) OR (training[Title/Abstract]))) AND (patient[Title/Abstract]) AND (caregiver[Title/Abstract])) AND ((((((((((sleep) OR (nutrition)) OR (physical activity)) OR (mood)) OR (social life)) OR (flare up)) OR (medications)) OR (brochure)) OR (video)) OR (cognitive)) OR (behavioural))	148	6
**N- Non-pharmacological approach**	((pain[Title/Abstract]) AND (chronic pain[Title/Abstract])) AND ((((((((((spinal cord stimulation[Title/Abstract]) OR (SCS[Title/Abstract])) OR (radiofrequency[Title/Abstract])) OR (intrathecal drug delivery device[Title/Abstract])) OR (IDDD[Title/Abstract])) OR (intrathecal drug delivery system[Title/Abstract])) OR (acupunture[Title/Abstract])) OR (neurolysis[Title/Abstract])) OR (physical activity[Title/Abstract])) OR (psychotherapy[Title/Abstract])) OR (music therapy[Title/Abstract]))	2426	31
**T- Team**	(((pain[Title/Abstract]) AND (chronic pain[Title/Abstract])) AND (team[Text Word])) AND ((((interdispiplinary) OR (multidisciplinary)) OR (approach)) OR (telemedicine))	625	5
**TOTAL**		5250	83

Table S2. Articles cited in 'Patient's perception' (P).

Study and Year	Design	Partecipants	Contributions
Campbell J.N. et al. 1995 [5]	Review	America Pain Society Presidential address.	Aim and targets to manage pain in the society and in the hospital
Gibson S. J. Et al. 2001 [6]	Review	The author summarized the evidence derived from 156 articles between the 1980 and 2000.	There are important differences in patient's perception depending by age and sex.
Lautenbacher S. et al. 2017 [7]	Systematic Review and Metanalysis	31 studies on pain threshold and 9 studies assessing pain tolerance threshold.	Pain threshold increases with age, while pain tolerance thresholds did not show substantial age-related changes.
Ahmed Y. et al. 2017 [8]	Systematic Review and Metanalysis	13 observational and RCT studies on gender-specific pain intensity in cancer patients.	Baseline perceived pain intensity in cancer patients did not significantly differ based on gender.
Fillingim R.B. et al. 1995 [9]	Systematic Review	35 observational and RCT studies on gender-specific pain intensity in cancer patients.	The literature indicates that females exhibit greater sensitivity to noxious stimulation than males, and there may be evolutionary advantages for these gender differences in pain regulation.
Racine M. et al. 2012 [10]	Systematic Review	A total of 172 articles published between 1998 and 2008 were retrieved, analyzed, and synthesized.	This review suggests that females (F) and males (M) have comparable thresholds for cold and ischemic pain, while pressure pain thresholds are lower in F than M. There is strong evidence that F tolerate less thermal (heat, cold) and pressure pain than M but it is not the case for tolerance to ischemic pain, which is comparable in both sexes.
Riley J.L. et al. 1998 [11]	Systematic Review and Metanalysis	34 studies analyzed.	This study compels to caution authors to obtain adequate sample sizes and hope that this meta-analytic review can aid in the determination of sample size for future studies.
Casale R. et al. 2021 [12]	Review	An Expert Group was convened to review the literature on pain conditions that are overrepresented in women.	Women are affected by pain diseases more frequently and severely than men and that they report pain more frequently and with a lower pain threshold than men

Fillingim R.B. et al. 1999 [13]	RCT	This experiment examined recent clinical pain as well as thermal pain thresholds and tolerances in 209 (117 female, 92 male) healthy young adults.	The experimental pain responses may be more clinically relevant for females than males.
Fillingim R.B. et al. 2000 [14]	RCT	This experiment examined recent pain complaints and laboratory pain responses as a function of sex and reported family history of pain in 212 (122 female, 90 male) young adults	A positive family history of pain was associated with increased reports of pain over the previous month and poorer general health as well as enhanced sensitivity to thermal stimuli among females but not males.
Coghill R.C. et al. 2003 [15]	RCT	A total of 17 normal, healthy subjects (8 women and 9 men) participated in this study. All participants were white and ranged in age from 21 to 40 years old (mean age 26 years old).	Using psychophysical ratings to define pain sensitivity and functional magnetic resonance imaging to assess brain activity, the authors assessed that highly sensitive. Individuals exhibited more frequent and more robust pain-induced activation of the primary somatosensory cortex, anterior cingulate cortex, and prefrontal cortex than did insensitive individuals.
Stroemel-Scheder C. et al. 2020 [16]	Systematic Review	29 studies were included in this review.	A reset of enhanced pain sensitivity and vulnerability following Recovery Sleep, especially when total Sleep Deprivation was implemented, and pressure pain or painful symptoms (human studies) were found.
Torensma B. et al. 2016 [17]	Systematic Review	From a total of 1818 identified studies, seven studies fulfilled the inclusion criteria, whereby only one study tested the pain threshold difference between obese and non-obese.	There is a tendency towards higher pain thresholds in obese subjects. Neither substantial weight loss, nor gender, were factors explaining difference in threshold
Price R. C. et al. 2013 [18]	RCT	A total of 20 obese participants [10M/10F, BMI mean (SD) = 41.5 kg/m2 (9.4 kg/m2)] and 20 age- and gender-matched non-obese controls [10M/10F, BMI mean (SD) = 23.5 kg/m2 (2.9 kg/m2)] were studied.	Obese participants are less sensitive than non-obese individuals, but only on areas with excess subcutaneous fat.
Merskey H. et al. 1994 [19]	Perspective Review	The author assessed 41 articles dated between 1965 and 1990 to explore the importance of behaviour and language starting by the IASP definition of pain:' an unpleasant sensory and emotional experience associated with actual or potential tissue damage, or described in terms of such damage.'.	The behaviour and the language could affect the perception of pain. The author tried to give a classification and definition of pain.

RCT Randomized controlled trial

Table S3. Articles cited in ‘Assessment’ (A).

Study and Year	Design	Partecipants	Contributions
Raja S. N. et al. 2020 [20]	Narrative review	This review provides a synopsis of the critical concepts, the analysis of comments from the IASP membership and public, and the committee’s final recommendations for revisions to the definition and notes, which were discussed over a 2-year period.	The task force ultimately recommended that the definition of pain be revised
Dworkin R.H. et al. 2008 [21]	Narrative review	Systematically collecting and reporting the recommended information needed to evaluate the clinical importance of treatment outcomes of chronic pain clinical trials	It is recommended that 2 or more different methods be used to evaluate the clinical importance of improvement or worsening for chronic pain clinical trial outcome measures
Glowacki D. et al. 2015 [22]	Narrative review	Use of recommended evidence-based practices in pain management and assessment	Useof interdisciplinary pain teams can lead to improvements in patients’ pain management, pain education, outcomes, and satisfaction.
Filingim R. B. et al. 2016 [4]	Narrative review	This article discusses assessment of chronic pain, including approaches available for assessing multiple pain domains and for addressing pathophysiological mechanisms. We conclude with recommendations for optimal pain assessment	Systematic pain assessment will improve pain diagnoses by reducing observer bias, which is more likely to emerge in the absence of carefully collected data regarding the patient’s pain.
Balagué F. et al. 2012 [23]	Narrative review	Searches on non-specific low back pain covered the years 2007–10.	Both patient preferences and clinical evidence should be considered for pain management, but generally self-management, with appropriate support, is recommended and surgery and overtreatment should be avoided.
Breivik H. et al. 2008 [24]	Narrative review	Review of acute and chronic pain assessment tools.	Any assessment of pain must consider other factors, such as cognitive impairment or dementia, and assessment tools validated in the specific patient groups being studied.
Burbank K. M. et al. 2008 [25]	Narrative review	Chronic shoulder pain evaluation and diagnosis	Review of chronic shoulder pain.
Morone N.E.et al. 2013 [26]	Author manuscript	To evaluate pain in patients as exemplified. by the fifth vital sign has exposed serious deficits in practitioner education and training in pain assessment and management.	Expanding pain education and training is critical to remedying some of the issues the routine report of pain by patients has uncovered.

Breivik H. et al. 2000 [27]	Prospective non-randomized trial	To examine agreement and estimate differences in sensitivity between pain assessment scales.	In this acute pain model, the VRS-4 was less sensitive than the VAS. The simulation results demonstrated similar sensitivity of the NRS-11 and VAS when comparing acute postoperative pain intensity. The choice between the VAS and NRS-11 can thus be based on subjective preferences.
Attal N. et al. 2011 [28]	RCT	Patients (n = 132) from 11 French multidisciplinary pain or rheumatology centres were classified by a first investigator into 4 groups derived from the Quebec Task Force<br>Classification of Spinal Disorders (QTFSD).	This study confirms the psychometric properties of the DN4 questionnaire to assess neuropathic pain in patients with low back pain

RCT Randomized controlled trial; VAS visual analogue scale; NRS numeric rate scale

Table S4. Articles cited in 'Tailored approach' (T).

Study and Year	Design	Partecipants	Contributions
Abramoff B. et al. 2020 [29]	Author manuscript	Summarize methods for management of the osteoarthritis	Osteoarthritis pathology, diagnosis, and treatment options.
Cuomo A. et al. 2019 [30]	Mini review	Mini review of 25 articles.	A new model termed "trolley analgesic model" that will allow adopting tailored therapies with dynamic multimodal approaches for pain management according to 1) the pain intensity, 2) the physiopathology of pain, 3) the complexity of symptoms, 4) the presence of comorbidity, and 5) the physio pathological factors and the social context.
Gelot S. et al. 2014 [31]	Author manuscript	Review of the guideline, empirical protocols, and the science underlying opioid rotation and tritration.	Methods to manage opioids in patients with organ impairment.
Knotkova H. et al. 2009 [32]	Review	Review of quasi-randomized and RCTs.	The equianalgesic dose table remains a cornerstone of published guidelines for opioid rotation, a review of the science and the clinical practices that have evolved around the use of the table demonstrates substantial limitations.
Moseley G. L. et al. 2004 [33]	RCT	Up-to-date review of the pharmacological treatment of neuropathic pain with emphasis on the latest evidence-based recommendations for its pharmacological treatment.	Change in pain cognitions is associated with change in physical performance, even when there is no opportunity to be physically active. Unhelpful pain cognitions should be considered when interpreting physical assessments.
Attal N. et al 2009 [34]	Review	Establish best practices for opioid rotation.	The authors provide some statements for the treatment of the chronic pain. Drugs proposed as first line include tricyclic antidepressants (particularly amitriptyline), serotonin–norepinephrine reuptake inhibitors (particularly duloxetine), pregabalin and gabapentin. Second line treatments include lidocaine plasters and capsaicin high concentration patches for peripheral neuropathic pain only, and tramadol. Third line treatments include strong opioids and botulinum toxin A (for peripheral neuropathic pain).

Fine P. G. et al. 2009 [35]	Expert panel	A literature search was performed using PubMed, MEDLINE, and the Cochrane Library, using a PRISMA flowchart.	Evidence based recommendations have recently been updated for the pharmacotherapy of neuropathic pain. Recent progress in the diagnosis, assessment, and understanding of its mechanisms offers the perspective of a more rational therapeutic management, which should result in better outcome.
Feng. X. Et al. 2017 [36]	Systematic Review	Systematic review of RCTs and pharmacological studies about drugs involved in the management of the pain.	Effective and safe combination therapy of opioids can be achieved by promoting the awareness of potential changes in therapeutic efficacy and toxicities, prescribing alternatives, or changing administration strategy, tailoring dose, reviewing the appropriateness of orders, and paying attention to medication monitoring.
Ayad. S. et al. 2019 [37]	Narrative review	Narrative review about respiratory depression related to opioid in the post-operative period.	New tools and technologies currently under development are expected to improve the prediction of respiratory depression especially in patients requiring opioids to alleviate acute postoperative pain.
Sporer. K.A. et al 2019 [38]	Review	Narrative review about serotonin syndrome.	This syndrome probably has a low incidence, even among patients taking these drug combinations, and there is likely to be some other yet unidentified inciting factor causing some patients to develop a full serotonin syndrome.
Jacox A. et al. 1994 [39]	Guidelines	New clinical practice guidelines for the management of pain in patients with cancer	Assessment and approach to the chronic cancer pain and its management.
Olarte J.M.N. et al. 2017 [40]	Good clinical practice of Spain	Breakthrough cancer pain and rational drug use	It is important to make more easier the access to opioids for patient affected by cancer and pain.
Vega-Loza A. et al. 2020 [41]	Commentary	Gene therapies to reduce chronic pain: are we there yet?	Studies that have correlated the contribution of genes to the pain process and novel therapeutic approaches to relieve pain have paved the way for a new era of potential alternative treatments. CRISPRi-KRAB and ZF-KRAB show great promise because of their high specificity, ability to target genes at the DNA level and long-lasting effects. However, before these precision medicine therapies are accessible to the public, gene therapy manufacturing improvements, a better understanding of the immune responses and a clear regulatory pathway will be needed.

RCT: Randomized controlled trial

Table S5. Articles cited in 'Iterative evaluation' (I).

Study and Year	Design	Partecipants	Contributions
Mucke M. et al. 2018 [42]	Review	We found 16 studies involving 1750 people. Studies lasted 2 to 26 weeks.	All cannabis-based medicines pooled together were better than placebo for the outcomes substantial and moderate pain relief and global improvement. All cannabis-based medicines pooled together were better than placebo in reducing pain intensity, sleep problems and psychological distress (very low- to moderate-quality evidence). There was no difference between all cannabis-based medicines pooled together and placebo in improving health-related quality of life, stopping the medication because it was not effective, and in the frequency of serious side effects (low-quality evidence). Herbal cannabis was not different from placebo in reducing pain and the number of people who dropped out due to side effects (very low-quality evidence).
Karran E.L. et al. 2020 [43]	Systematic Review and Metanalysis	We included studies involving adult participants that evaluated relationships between one or more of the SDH and CLBP frequency or LBP outcomes (beyond 3 months).	Our findings suggest that greater recognition of the contribution of SDH to disparities in LBP outcomes is warranted and this has the potential to usefully inform strategies to impact burden.
Emerick T. et al. 2020 [44]	Commentary	This commentary will use a bullet point format to present combined expert opinions.	A figure that shows when is appropriate, probably appropriate, and inappropriate to use telemedicine.
Cascella M. et al. 2021 [45]	Review	This article is based on the direct experience of a group of clinicians and attempts to provide a framework to prepare physicians, patients with chronic pain, and caregivers to use telemedicine.	This approach can deliver tailored pain management, providing improved access to health services and creating and maintaining a therapeutic alliance in the long term.

RCT Randomized controlled trial

Table S6. Articles cited in ‘Education’ (E).

Study	Year	Design	Partecipants	Contributions
Brain K et al. 2018 [46]	2018	Systematic Review	23 studies were included and assessed in a metanalysis.	The nutrition therapy is safe and could be helpful in the management of the chronic pain.
Roberts M et al. 2016 [47]	2016	Observational Prospective	101 patients (44 males and 57 females) completed a questionnaire that investigate multiple functional domains.	Sleep and pain are strictly related, and it is important to explore and educate the sleeping sphere of the patient to improve his management of chronic pain.
Geneen LJ et al. 2017 [48]	2017	Systematic Review of reviews	21 reviews were included with a total of 37,143 participants.	Even if the studies that investigate physical therapy are few and the follow-up is not enough, the physical activity and daily exercise reduce the adverse events that increase the pain.
Rantonen et al. 2013 [49]	2013	RCT	181 participants were equally divided in two groups: one group received both Back Book, a pamphlet that explain the strategies to fight the low back pain, and a face-to-face dialogue with a formed nurse; the second group received only the pamphlet. The authors assessed the RM-18, 15-qol, and VAS score for four years.	The combination treatment of book and dialogue is not superior to the pamphlet alone.
Manar AA et al. 2014 [50]	2014	Systematic Review	20 studies about behavioural changes and video-assisted therapy during chronic pain were assessed.	Videos that show real people doing something are more effective than videos that provide only spoken or graphical information.
Chi N-C et al. 2020 [51]	2020	Systematic review	25 studies about the following therapies were evaluated: educational interventions, cognitive-behavioural interventions, and technology-based-	Educational and cognitive-behavioural interventions improve the family caregivers’ and patients’ outcome. Technology-based interventions are a valid solution to support the family caregivers in pain management.

RCT Randomized controlled trial RM-18 Roland-Morriss disability scale 15-qol 15 items quality of life questionnaire VAS visual analogue scale

Table S7. Articles cited in 'Non-pharmacological approach' (I).

Study	Design	Setting	Contributions
Kumar K et al. 2013 [52]	Retrospective cost-effectiveness study	313 patients underwent a SCS trial implant over 20 years.	Significant cost savings can be achieved with the use of SCS therapy compared with CMM in the management of PSPS, CRPS, PAD and RAP.
Echeverria-Villalobos M et al. 2021 [53]	Narrative review	Broad search of literature between January 2000 and July 2020.	SCS programming may mitigate neuroinflammation through modulation of WDR neurons and glial cell signalling, improving clinical and functional outcomes in patients with chronic lumbar pain and PSPS.
Chakravarthy K et al. 2019 [54]	Review	Multiple database queries and recent peer-reviewed conference presentations.	Burst-SCS likely provides pain relief via multiple mechanisms at the level of both the spinal cord and the brain. In particular, it seems to modulate medial spino–thalamo–cortical pathways which are responsible for the emotional and affective parts of painful sensation.
Sdrulla AD et al. 2018 [55]	Author manuscript	Broad search of literature.	SCS represents a safe and effective therapy; it seems to act also through supraspinal mechanism of stimulation of the inhibitory descending pathways; further research is needed to investigate its mechanisms and to improve its applications.
Van Boxem K et al. 2010 [56]	Narrative review	Broad search of literature until to December 2009.	When conservative treatment fails: in (sub)acute lumbosacral radicular pain under the L3 level because of a contained herniation, transforaminal corticosteroid administration is recommended; in chronic lumbosacral radicular pain, PRF treatment at the level of the spinal ganglion can be considered; for refractory lumbosacral radicular pain, adhesiolysis and epiduroscopy can be considered; in patients with a therapy-resistant radicular pain in the context of an FBSS, spinal cord stimulation is recommended.
Aryal et al. 2021 [57]	Sistematic review	Systematic selection of 37 studies in Google Scholar, PubMed Central, and Web of Science.	SCS therapy has shown great promise in treating patients who are affected by refractory chronic low back pain or not eligible for surgery.
Dones I et al. 2018 [58]	Review	Broad search of literature.	Conventional SCS may be considered as an effective, safe, well-tolerated and reversible treatment option for severe drug-refractory neuropathic pain in accurately selected patients.
Kumar K et al. 2007 [59]	Multicentre RCT	100 FBSS patients with neuropathic radicular leg pain were randomized to receive SCS plus CMM (SCS group) or CMM alone (CMM group) for at least 6 months.	The SCS group experienced improved leg and back pain relief, quality of life, and functional capacity, as well as greater treatment satisfaction, compared with CMM alone.

North RB et al. 2005 [60]	RCT	50 patients selected for reoperation by standard criteria after lumbosacral spine surgery were equally randomized to receive SCS or reoperation.	SCS is more effective than reoperation as a treatment for persistent radicular pain after lumbosacral spine surgery, with decreased opiate analgesics use.
Pollard EM et al. 2019 [61]	Systematic Review and Meta-analysis	12 comparative RCTs including a total of 489 patients with chronic back and/or limb pain of greater than one year duration were included.	In patients with refractory spine/limb pain, SCS was associated with increased odds of reducing pain medication consumption, when compared with medical therapy; clinical significance between different types of SCS requires further study.
Kapural L et al. 2016 [62]	Multicentre RCT	171 patients affected by back and/or leg pain (VAS >5 cm) completed a 24-month follow-up after randomly treatment with HF10-SCS or low-frequency SCS, in order to evalue the responder rate, defined as ≥50% back pain reduction from baseline at 3 months with a secondary end point at 12 and 24 months.	This study demonstrates long-term superiority of HF10 therapy compared with traditional SCS in treating both back and leg pain.
Deer T et al. 2018 [63]	RCT	100 subjects with chronic pain of the trunk and/or limbs were randomized to receive traditional tonic stimulation or burst stimulation for the first 12 weeks, and then the other stimulation mode for the next 12 weeks.	The SUNBURST study demonstrated that burst spinal cord stimulation is safe, effective, preferred by patients and superior to tonic stimulation for the treatment of chronic pain.
Odonkor C et al. 2020 [64]	Retrospective clinical trial	Data of 174 patients undergoing SCS trials were retrospectively collected. Trial duration ranged from 3 to 14 days. Pain scores were obtained prior to, and at the end of the trial period.	Paresthesia-based tonic stimulation (but not HF10 and Burst SCS), younger age, and surgical spine history have significant effects on successful SCS trials. However further research on HF10 and burst stimulation was needed due to their effects on affective and emotional aspects of pain pathways.
Yang S et al. 2020 [65]	Narrative review	58 studies were included after a PubMed search for papers published until August 20, 2019, that used PRF to treat pain resulting from spinal disorders.	PRF stimulation treatment could be an effective treatment for cervical and lumbar radicular and facet joint pain, without serious complications. Evidence on the effectiveness of PRF treatment for other spinal disorders is still lacking.

Erdine S et al. 2009 [66]	Pre-clinical trials	PRF is applied to the afferent axons of the sciatic nerves of rats, then the ultrastructure of the treated axons is observed after 10 days by electron microscopy, and it is compared to a control sham application to the contralateral sciatic nerve.	The internal ultrastructural components of the axons show microscopic damage after PRF exposure, including abnormal morphology of mitochondria, and disruption and disorganization of microfilaments and microtubules. The damage appears to be more pronounced for C-fibers than for A-delta and A-beta fibers.
Hagiwara S et al. 2009 [67]	Pre-clinical trial	Sciatic nerve of rats with induced inflammatory pain were treated with PRF, RF or sham treatment, followed by intrathecal administration of alpha2 and 5-HT3 receptor antagonist	The results suggest that the analgesic action of PRF involves the enhancement of noradrenergic and serotonergic descending pain inhibitory pathways.
Chang MC et al. 2018 [68]	Narrative review	63 studies were included after a PubMed search for papers published until August 31, 2017, that used PRF to treat peripheral neuropathic pain.	For radicular pain from spinal diseases, the evidence supports that PRF is an effective treatment. Similarly, PRF appears to be effective for postherpetic neuralgia and occipital neuralgia. On the other hand, for trigeminal neuralgia, PRF seems to be not appropriate and less effective than conventional RF. Data on the use of PRF for pudendal neuralgia, meralgia paresthetica, carpal tunnel syndrome, tarsal tunnel syndrome, and Morton's neuroma, is lacking.
Ke M et al. 2013 [69]	RCT	96 patients with thoracic (T2-11) PHN were equally randomized assigned into PRF or sham group. The treatment was done once a week for 3 weeks and its effects were evaluated at days 3, 7, and 14, and at months one, 2, 3 and 6 after PRF.	The angulus costae as PRF puncture target is an effective and safe therapeutic alternative for thoracic PHN treatment. Benefits include that the procedure is minimally invasive, provides short-term pain relief (until 6 months), improves quality of life, and reduction of tramadol dosage after treatment.

Pi ZB et al. 2015 [70]	RCT	128 patients with lower back or anterior abdominal wall acute PHN were randomly divided into two groups: Group A- oral treatment only with gabapentin, celecoxib and amitriptyline; group B- above oral therapy plus PRF using the paravertebral puncture US-guided technique.	US-guided spinal nerve posterior ramus PRF treatment of lower back or anterior abdominal wall PHN proved effective by reducing morphine use and improving sleep quality with fewer adverse reactions.
Kim ED et al. 2017 [71]	Retrospective comparative study	42 patients' medical records were analyzed: NRS and medication dose before and after 1 to 6 months after continuous epidural block and DRG PRF were evaluated.	DRG PRF was more effective than a continuous epidural block in treating zoster-acute-related pain and it may be a useful option for reducing the progression of neuropathic changes.
Kapural L et al. 2020 [72]	Review	Broad search of literature about cooled radiofrequency for knee osteoarthritis management.	Cooled radiofrequency ablation has shown clinical effectiveness in managing knee OA pain, with a majority of patients experiencing upwards of 12 months of analgesic effect.
Carvajal et al. 2018 [73]	Observational study	Follow-up observational study designed to evaluate 11-year results of IDDS for refractory pancreatic cancer pain in 93 patients at the Institut de Cancérologie de L'Ouest, Paul Papin in France.	IDDS for refractory malignant pain due to pancreatic cancer was associated with pain relief with a significant statistical difference between preimplantation NRS pain score and 1 week, 1 month, and 3 months. In addition, IT administration of a lower dosage of drug to obtain the same analgesic action compared to other routes reduces side effects.
Capozza MA et al. 2021 [74]	Narrative review	A narrative overview of the literature has been performed, synthesizing the findings of literature from 1986 to 2021.	Intrathecal route administration of medication appears to be a good way to treat chronic refractory pain. However it requires good clinical practice, adherence to guidelines and continuous improvement in clinical technology.
Deer TR et al. 2017 [75]	Review PACC guidelines	Extensive literature search identified publications between January 15, 2007 and November 22, 2015 and authors contributed additional relevant sources about intrathecal therapy.	New algorithms and guidance have been established to improve care and suitability of the use of intrathecal drug delivery.

Deer TR et al. 2011 [76]	Meta-analysis	Clinician research leaders in pain medicine outline consensus guidelines for the implementation of intrathecal therapy in patients with cancer-related pain and other end of life states causing pain.	These consensus guidelines are intended to assist clinicians in identifying the candidacy of patients with careful consideration of their medical comorbidities and prior therapies, communication with the oncologist, proper psychological evaluation, and appropriate trailing technique.
Eide PK et al. 1998 [77]	Clinical study	Quantitative examination of sensory and pain perception was performed in the trigger area and the contralateral nonpainful facial skin area for 39 patients with trigeminal neuralgia who had been previously treated with PRGR and for 14 non-surgically treated patients.	Relief of pain after PRGR depends on the normalization of abnormal temporal summation of pain, which is independent of general impairment of sensory perception.
Resnick DK et al. 1998 [78]	Retrospective clinical trial	A retrospective chart review was conducted to determine the efficacy of MVD during a 1 year follow-up after the treatment of TGN in 22 patients in whom the onset occurred during childhood and who underwent exploration of the cerebellopontine angle.	At the time of their last follow-up, 9 of these patients (43%) continued to have complete pain relief. Patients whose symptoms begin in childhood do not enjoy the same therapeutic response to MVD as do patients with TGN onset in adulthood, maybe related to an increased incidence of venous compression.
Sharma R et al. 2018 [79]	Meta-analysis	5 prospective comparative trials were selected to evaluate the efficacy or complications of MVD and stereotactic radiosurgery for medically refractory trigeminal neuralgia.	MVD seems to be more efficacious than GKT as a first line treatment for trigeminal neuralgia immediately as well as on a long term basis, with lower rate of facial numbness and dysesthetic pain.
Wang YJ et al. 2016 [80]	Review	Broad search of literature.	Percutaneous balloon compression, glycerol rhizotomy, and radiofrequency thermocoagulation offer immediate and durable pain relief, with relatively low, but variable rates of complications.
Xu-Hui W et al. 2011[81]	Retrospective clinical study	3370 patients undergoing PRGR between 1983 and 2003 were followed up for 13.1 years and the outcomes were investigated.	PRGR is a minimally invasive and simple first-line surgical modality yielding a favourable outcome for trigeminal neuralgia unresponsive to pharmacotherapy. The major complication was reversible facial numbness, among which hypesthesia outweighed hypalgesia.
Pradel W et al. 2002 [82]	Clinical study	A newly developed cryoprobe for peripheral nerves was used in 19 patients to freeze the infraorbital nerve or the inferior alveolar nerve.	Cryosurgery widens the range of repeatable methods available to treat trigeminal neuralgia, giving the patients a free-pain period of at least 6 months.

He T at al. 2015 [83]	Review	Broad search of literature.	Acupuncture seems to lead to long-term decreases in pain with multiple effects on the central and peripheral nervous systems. Functional magnetic resonance imaging has shown the involvement of neuron clusters releasing endogenous opioids, serotonin, and norepinephrine, which may have downstream effects on nociceptors, inflammatory cytokines, and other physiologic pain pathways
Vickers AJ et al. 2018 [84]	Systematic review and meta-analysis	39 RCTs about acupuncture, for a total of 20,827 patients, searched in MEDLINE and the Cochrane Central Registry until December 31, 2015.	Acupuncture for the treatment of chronic pain was superior to sham as well as no acupuncture control for each pain condition. The effects of acupuncture persist over time with only a small decrease, approximately 15%, in treatment effect at 1 year, and it cannot be explained solely in terms of placebo effects.
Corbett MS et al. 2013 [85]	Systematic review and meta-analysis	114 RCTs comparing the effectiveness of acupuncture with other relevant physical treatments for alleviating pain due to knee osteoarthritis in 9,709 patients were included after a comprehensive search until January 2013.	8 intervention, interferential therapy, acupuncture, TENS, pulsed electrical stimulation, balneotherapy, aerobic exercise, sham acupuncture, and muscle-strengthening exercise, produced a statistically significant reduction in pain when compared with standard care, with acupuncture being considered as one of the more effective in a sensitive analysis. However, much of the evidence in this area of research is of poor quality.
Deare JC et al. 2013 [86]	Systematic review and meta-analysis	9 trials (395 participants) evaluating any type of invasive acupuncture for fibromyalgia were included.	There is low to moderate-level evidence that compared with no treatment and standard therapy, acupuncture (in particular electric one) safely improves pain and stiffness in people with fibromyalgia, but not in reducing pain or fatigue, or improving sleep or global well-being.
Kelly RB et al. 2019 [87]	Review	Broad search of literature.	Multiple factors may contribute to variability in acupuncture's therapeutic effects, including needling technique, number of needles used, duration of needle retention, acupuncture point specificity, number of treatments, and numerous subjective (psychological) factors. Acupuncture may provide modest benefits in the treatment of chronic low back pain, tension headache and chronic headache, migraine headache prophylaxis, and myofascial pain. Acupuncture treatment has a notable placebo response, or meaning response, that may be responsible for much of its demonstrated benefits.
Ettinger WH et al. 1997 [88]	RCT	A randomized, single-blind clinical trial lasting 18 months conducted at 2 academic medical centers, including a total of 439 community-dwelling adults aged 60 years or older, with radiographically evident knee osteoarthritis, pain, and self-reported physical disability, who are divided into an. aerobic exercise program, a resistance exercise program, and a health education program.	Older disabled persons with osteoarthritis of the knee had modest improvements in measures of disability, physical performance, and pain from participating in either an aerobic or a resistance exercise program. These data suggest that exercise should be prescribed as part of the treatment for knee osteoarthritis.

Ferreira RM et al. 2019 [89]	Systematic review and meta-analysis	5 of 39 RCTs were included in meta-analysis with the aim of evaluate the effectiveness of the non-surgical and non-pharmacological interventions commonly used for knee osteoarthritis (OA) patients.	Exercise is the best intervention for knee OA patients. Pulsed Electromagnetic Fields and Moxibustion showed to be the most promising interventions from the others options available.
Cifu DX et al. 2021 [90]	Book	Broad search of literature.	An essential guide for the entire rehabilitation team.
Bradt J et al. 2016 [91]	Systematic review and meta-analysis	52 RCTs published from their inception to January 2016, with a total of 3731 participants, were included to evaluate music therapy interventions offered by trained music therapists, as well as music medicine interventions, which are defined as listening to pre-recorded music, offered by medical staff, for improving psychological and physical outcomes in adult and pediatric patients with cancer.	Music therapy interventions may have beneficial effects on anxiety, pain, fatigue and QoL in people with cancer. Furthermore, music may have a small effect on heart rate, respiratory rate and blood pressure. Most trials were at high risk of bias.

RCT Randomized controlled trial SCS = Spinal Cord Stimulator, CMM = Common Medical Management PSPS = Persistent Spinal Pain Syndrome CRPS = Complex Regional Pain Syndrome, PAD = Peripheral Arterial Disease, RAP = Refractory Angina Pectoris, WDR = Wine Dynamic Range neurons, PRF= Pulsed Radiofrequency, FBSS = Failed Back Surgery Syndrome, RCT= Randomized Controlled Trials, VAS = Visual Analogue Scale, HF10 = High Frequency SCS, SUNBURST = Success Using Neuromodultion with Burst, PHN = Post-Herpetic Neuralgia, US = Ultrasonography, DRG = Dorsal Root Ganglion, NRS = Numeric Rating Scale, OA = Osteoarthritis, IDDS = Intrathecal Drug Delivery System, IT = Intrathecal, PRGR = Percutaneous Retrogasserian Glycerol Rhizotomy, MVD = Microvascular Decompression, TGN = Trigeminale Neuralgia, GKT = Gamma Knife Thalamotomy, TENS = Transcutaneous Electric Nerve Stimulation, QoL = Quality of LifeCMM

Table S8. Articles cited in 'Team' (T).

Study	Design	Setting	Contributions
Gauthier K et al. 2019 [92]	Review	Update of CADTH Rapid Response report.	A multidisciplinary approach delivered by at least two healthcare professionals of different backgrounds allows optimal assessment and management of chronic non-malignant pain, with significant improvements from baseline in pain and function or disability.
Marra A et al. 2018 [93]	Review	Broad search of literature.	Polypharmacy and inappropriate prescribing of medications in older persons may lead to a significant risk of adverse drug-related events and mortality. Therapeutic reconciliation is recommended at every transition of care to improve appropriateness of prescription.
Schwan J et al. 2019 [94]	Review	Broad search of literature.	A multidisciplinary approach is preferable for chronic pain treatment in older adults, including multimodal medications, selected interventions, physical therapy and rehabilitation, and psychological treatments
Blair MJ et al. 2015 [95]	RCT	241 veterans of Iraq and Afghanistan with chronic and disabling musculoskeletal pain of the cervical or lumbar spine or extremities were enrolled from December 20, 2007, through June 30, 201. 121 patients receive the stepped-care intervention (12 weeks of analgesic treatment followed by 12 weeks of cognitive behavioural therapy) and 120 patients receive usual care.	A stepped-care intervention that combined analgesics, self-management strategies, and brief cognitive behavioural therapy resulted in statistically significant reductions in pain-related disability, pain interference, and pain severity in veterans with chronic musculoskeletal pain.
Santini A et al. 2021 [96]	Systematic Review	24 quantitative studies regarding multimodal therapeutic approaches of chronic musculoskeletal pain in the veteran population were included after a comprehensive systematic review of the literature on Cochrane Library, PubMed, CINAHL e PsycINFO databases was conducted, from 2001 to 2020.	Multimodal therapeutic approaches seem to guarantee a good management chronic musculoskeletal pain and related mental disorders, and the reduction and control to opioid use.

RCT randomized controlled trial
